# The Citadel of the Senses

**Published:** 1986-10

**Authors:** M. G. Wilson


### Book Reviews

### THE CITADEL OF THE SENSES

**MacDonald Critchley**
**Raven Press, New York.**

When your editor received this elegant and beautifully bound volume for review it was his intention to do the honorable thing and pass this book by an eminent neurologist (and graduate of Bristol University) to a neurological colleague for his informed opinion on it. Before actually doing so however he began to read it, became immediately captivated and so decided to exercise his *droit du seigneur* and review it himself. It is not, as a matter of fact, a book for the specialist but for the general medical reader, even, in these days of obsession by the general public with anything medical, for the general reader. The author in his preface describes it as a collection of jottings, sharing memories of personalities and events of an earlier, prescientific era of neurology. The opening essay is on 'The nose as the sentinel of the citadel of the senses'. We are immediately in a literary rather than a scientific world, in the company of Pliny, Bunyan, Samuel Johnson and Mackenzie 'singing of smells, perfumes, odours, whiffs and sniffs, of aromas, bouquets and fragrances, and also, 'though only temporarily and restrainedly, I promise you', of effluvia, reeks, foetors, stenches and stinks.' Science creeps in from time to time and we learn that the power of some insects to detect others of their species more than a mile away can be equated with a Londoner sniffing out a female in Siberia. Soon we are back with Virginia Woolf, enjoying, through the nose of Elizabeth Barrett Browning's spaniel Flush, the olfactory delights of snifing her way through the streets and alleys of Florence. All irresistable reading, if you have a taste for that sort of thing and *your soul isn't fettered to the office stool.*

There are biographical essays on some great men of neurology. On Hughlings Jackson, the sage of Manchester Square, who became a legend in his lifetime. By a cruel irony, after only 11 years of happily married life his wife developed a terminal illness characterised by 'countless epileptiform seizures of a focal character, the significance of which was all too evident to her husband.' Though a scholar, he would not spend money on books, and, if he needed a neurological reference would borrow the book from a colleague, who after repeated requests for its return would find the relevant pages torn out. On Babinski, who described the sign for which he became renowned early in his career, and who made many other original contributions to medicine including a description of the adiposo-genital syndrome a year before Frohlich. On Newman Nield, his first chief, and an eccentric for whom he had great affection. He treated facial neuralgias with leeches, for intractable headache a seton was inserted into the nape of the neck and pulled every day until 'laudable pus' was produced and the victim protested that the headache was 'much better'. Of especial interest to Bristolians is an essay on John Addington Simmons, the father of Bristol General Hospital, 'that best type of physician who, inspired by Sir Thomas Browne, adorns the profession of medicine' and on his son, the man of letters, John Addington Symons Jr. 'At birth he was priveleged to receive the gift of gold, the frankincense of the intellect, the myrrh of artistic discrimination, but a wicked fairy darkened the scene. Her contributions were lifelong bodily and mental ill health and sexual inversion.' The extraordinary story of his academic brilliance, of his morbid imagination and of his homosexuality are told with clinical insight.

Perhaps these tit-bits may give something of the flavour of this book. Amongst other subjects for essays are 'Why do we laugh?', 'The Land of smiles', 'Oscar Wilde and his association with the West Country', 'The secret world of the singer', 'The inscrutability of Pain', the final essay is on 'Dr Samuel Johnson's Stroke . . .' told largely by Dr Johnson himself through his letters to friends.

I think this is a great book, worthy to become a classic, it is a treasure house and I enjoyed reading every word. Dr Chritchley writes from the commanding heights achieved in a long life rich in experience, and passes on to us something of his clinical wisdom and his special insights, illuminated from the wider world of literature. This is a book to possess and to re-read, and rare nowadays, it is worthily bound.

M. G. Wilson

